# The minimum detectable difference (MDD) and the interpretation of treatment-related effects of pesticides in experimental ecosystems

**DOI:** 10.1007/s11356-014-3398-2

**Published:** 2014-08-15

**Authors:** T. C. M. Brock, M. Hammers-Wirtz, U. Hommen, T. G. Preuss, H-T. Ratte, I. Roessink, T. Strauss, P. J. Van den Brink

**Affiliations:** 1 Alterra, Team Environmental Risk Assessment, Wageningen University and Research Centre, P.O. Box 47, 6700 AA Wageningen, The Netherlands; 2 Research Institute for Ecosystem Analysis and Assessment (gaiac), Kackertstrasse 10, 52072 Aachen, Germany; 3 Fraunhofer Institute for Molecular Biology and Applied Ecology (IME), Auf dem Aberg 1, 57392 Schmallenberg, Germany; 4 Institute for Environmental Research, RWTH Aachen University, Worringer Weg 1, 52074 Aachen, Germany; 5 Department of Aquatic Ecology and Water Quality Management, Wageningen University, PO Box 47, 6700 AA Wageningen, The Netherlands; 6 Present Address: Bayer CropScience, Monheim, 6690 Germany

**Keywords:** Mesocosms, Microcosms, Environmental effect assessment, Experimental design, Statistical power, Population responses, Plant protection product

## Abstract

In the European registration procedure for pesticides, microcosm and mesocosm studies are the highest aquatic experimental tier to assess their environmental effects. Evaluations of microcosm/mesocosm studies rely heavily on no observed effect concentrations (NOECs) calculated for different population-level endpoints. Ideally, a power analysis should be reported for the concentration–response relationships underlying these NOECs, as well as for measurement endpoints for which significant effects cannot be demonstrated. An indication of this statistical power can be provided a posteriori by calculated minimum detectable differences (MDDs). The MDD defines the difference between the means of a treatment and the control that must exist to detect a statistically significant effect. The aim of this paper is to expand on the Aquatic Guidance Document recently published by the European Food Safety Authority (EFSA) and to propose a procedure to report and evaluate NOECs and related MDDs in a harmonised way. In addition, decision schemes are provided on how MDDs can be used to assess the reliability of microcosm/mesocosm studies and for the derivation of effect classes used to derive regulatory acceptable concentrations. Furthermore, examples are presented to show how MDDs can be reduced by optimising experimental design and sampling techniques.

**Electronic supplementary material:**

The online version of this article (doi:10.1007/s11356-014-3398-2) contains supplementary material, which is available to authorized users.

## Introduction to microcosm/mesocosm studies in environmental risk assessment

Microcosms and mesocosms are bounded test systems that are constructed artificially with samples from, or portions of, natural ecosystems or that consist of enclosed parts of natural ecosystems. These experimental ecosystems may be used as an ecological research tool for hypothesis testing and hypothesis generation (e.g. relating to food-web interactions) and in the environmental effect assessment of chemicals (e.g. to derive ecologically ‘safe’ levels of pollutants in surface water) (e.g. Caquet et al. 2000). Within the context of the registration of pesticides on the European market (EC 2009), it is a common practice to use microcosm/mesocosm experiments as a higher tier test approach to derive ‘regulatory acceptable concentrations’ (RACs) for edge-of-field surface waters (EFSA 2013). Also, for the derivation of environmental quality standards (EQSs) underlying the EU Water Framework Directive, microcosm/mesocosm tests may be used (Brock et al. 2006, 2011; EC 2011).

In environmental risk assessment procedures, the main advantages of microcosm/mesocosm studies over single-species laboratory tests and field monitoring studies are as follows: (i) better control over confounding factors, making it easier to demonstrate causality between exposures and ecological effects, (ii) the ability to replicate microcosm/mesocosm allowing the derivation of concentration–effect relationships and statistical interpretation of the treatment-related responses, (iii) the possibility to integrate more or less realistic exposure regimes of toxicants with the assessment of endpoints at higher levels of biological integration (e.g. population- and community-level responses), (iv) the possibility to study intra- and inter-species interactions and indirect effects within a community, and (v) the chance to perform medium- to long-term observations so that latency of effects and population and community recovery can be assessed.

To interpret the often complex ecological information and concentration–response relationships derived from microcosm/mesocosm experiments, it is a common practice to use univariate (e.g. Williams’ test, Kruskal–Wallis multiple comparison test and Dunnett’s test) and multivariate (e.g. Principal Response Curves and Monte Carlo permutation tests) statistical techniques to calculate no observed effect concentrations (NOECs) and lowest observed effect concentrations (LOECs) at the population or community level. The relevance of the information provided by these statistical tools is highly dependent on the test design of the microcosm/mesocosm experiment, particularly the number of test systems used as control and for each treatment, and the variability of the measurement endpoints between replicate test systems. In microcosm/mesocosm tests conducted for pesticide registration, the recommendation is to use an exposure–response experimental design with preferably five or more concentrations and at least two replicates per treatment and preferably a larger number of replicate test systems that serve as control (Giddings et al. 2002; OECD 2006a).

An issue that is frequently disputed is the statistical power of microcosm/mesocosm experiments to demonstrate effects at the population and community levels (e.g. Sanderson 2002; De Jong et al. 2005; Van den Brink 2006; EFSA 2013). Up until now, however, little practical guidance is available on ways to deal with the statistical power of a particular microcosm/mesocosm test and the related minimum detectable difference (MDD) for NOEC determination of relevant measurement endpoints, when evaluating microcosm/mesocosm tests. Furthermore, up to now, relatively few scientific publications reported and discussed MDDs for toxicity endpoints derived from microcosm/mesocosm tests (e.g. Hanson et al. 2003; Sanderson et al. 2009).

This paper discusses measures to optimise MDDs in designing and conducting microcosm/mesocosm experiments, as well as the use of MDDs in interpreting these semi-field tests for regulatory purposes.

## NOEC calculations and microcosm/mesocosm tests

In the statistical evaluation of concentration–response relationships observed in microcosm/mesocosm, it is a common practice to calculate NOECs for all measurement endpoints. Note that the potential sensitivity of different groups of water organisms may vary by several orders of magnitude, so that adopting a regression approach that allows EC_x_ values to be calculated for a broad range of water organisms may require testing a larger number of exposure concentrations than is practically feasible in experimental ecosystems. In addition, microcosm/mesocosm tests also aim to address both direct and indirect effects, and indirect effects may not follow a monotonous concentration–response relationship. Therefore, most ‘regulatory’ microcosm/mesocosm tests focus on environmentally realistic exposure concentrations (covering the PECs for different edge-of-field surface waters) and test significant deviations relative to controls rather than calculate EC_x_ values. Several methods are available to obtain this information (for an overview, see e.g. OECD 2006b).

In the examples presented in this paper, we used the multiple *t* test developed by Williams (1971, 1972) to calculate NOECs, primarily for direct effects but also for indirect effects characterised by a concentration–response relationship in the same direction, i.e. either a monotonous increase or decrease. This test is similar to the multiple *t* tests by Dunnett (1955, 1964) in comparing each treatment with the control, but in contrast to the Dunnett’s test, the Williams’ test assumes a monotonous concentration–response relationship. If data on the means per treatment are not monotonous, a moving average procedure is applied to achieve this. The assumption of a monotonous concentration–response is usually not violated when the treatment-related effect is directly caused by exposure to the pesticide (direct effect), but it can be questioned for responses that are caused by the interaction of direct and indirect effects resulting in a non-monotonous concentration–response relationship. For example, due to release of competition with a more sensitive competitor, the abundance of a species may increase at lower concentrations but its abundance may decrease at higher concentrations when toxic effects overrule the positive indirect effect (see e.g. Roessink et al. 2005). Possible non-monotonous concentration–response relationships may be better evaluated statistically using other multiple *t* tests like that of Dunnett (1955, 1964). However, when deriving RACs or EQSs from microcosm/mesocosm tests, the effect classification is mainly based on direct effects (e.g. EFSA 2013; Brock et al. 2011), and for an indirect effect to occur, there has to be a direct effect first. An additional advantage of the Williams’ test is its slightly higher power than the Dunnett’s test (Jaki and Hothorn 2013).

In order to achieve normal distribution and homogeneity of variance, abundance data are usually log-transformed for the statistical test. For the examples presented in this paper, we followed Van den Brink et al. (2000) using the transformation *y*(*x*) = ln(ax + 1), where *x* is the measured abundance and the factor ‘a’ is selected in such a way that the lowest non-zero abundance of the data set is transformed to 1.

## The MDD concept

The statistical reliability of the conclusions drawn from a microcosm/mesocosm test depends on the power of the test conducted, which in this case is the probability that the tests will find that a given difference between the means of a control and a treatment level is statistically significant. Power analysis can be used a priori to calculate the minimum number of replicates per treatment required so that one can be reasonably likely to detect a relevant effect of a given size for a given type I error level *α* and a given type II error level *β*. A priori power analysis of microcosm/mesocosm experiments may be difficult, given the inherent variability of these community-level test systems, e.g. due to stochastic events and variable environmental factors (like weather conditions) influencing species composition, food-web dynamics and fluctuations in population densities. For further details on statistical power analysis, we refer to Sokal and Rohlf (1995), Environment Canada (2005), OECD (2006b), Van der Hoeven (2008) and Sachs and Hedderich (2009). It is also possible to estimate an indicator of the statistical power of a microcosm/mesocosm test a posteriori: viz. the MDD. Synonyms of MDD are critical boundary (Sokal and Rohlf 1995) and minimum significant difference (Environment Canada 2005; Van der Hoeven 2008). The MDD defines the difference between the means of a treatment and the control that must exist in order to conclude that there is a significant effect (Environment Canada 2005). For the two-sample and multiple *t* tests, the MDD can be easily calculated by the rearranged formula of the *t* test, using Eqs. 1 or 2 when applying the treatment/control variances, *s* ^2^_0_ | *s* ^2^ in Eq. 1. In Eq. 2, *s* is the residual standard error (≡square root of the residual variance from a one-way ANOVA).

1 $$ \mathrm{MDD}={\left({\overline{x}}_0-\overline{x}\right)}^{*}={t}_{1-\alpha, df, k}\sqrt{\frac{s_0^2}{n_0}+\frac{s^2}{n}} $$

2 $$ \mathrm{MDD}={\left({\overline{x}}_0-\overline{x}\right)}^{*}={t}_{1-\alpha, df, k} s\sqrt{\frac{1}{n_0}+\frac{1}{n}} $$

where *t*_1 − *α*,*df*,*k*_ is the quantile of the *t*-distribution, *df* is the degrees of freedom, *k* is the number of comparisons, ${\left({\overline{x}}_0-\overline{x}\right)}^{*}$ corresponds to the difference between control and treatment mean and *n*_0_ and *n* are the sample sizes.

The MDD introduced above can only be derived from results of parametric tests, i.e. variants of the *t* test. In case the requirements of parametric tests (normal distribution, homoscedasticity) are not met and rank-based tests are appropriate (e.g. the Mann–Whitney *U* test), MDDs of medians between control and treatment can be computed (Van der Hoeven 2008), but this is much more laborious and is beyond the scope of the present paper. However, while the methodology used for parametric and non-parametric approaches may be different, the principal discussion and concept applies to both.

It has proved convenient to give the MDD as a percentage of the control mean (Eq. 3).

3 $$ \mathrm{MDD}\%=\raisebox{1ex}{$\mathrm{MDD}$}\!\left/ \!\raisebox{-1ex}{${\overline{x}}_0$}\right.\times 100 $$

As abundance data are usually log-transformed for statistical testing, the MDD is also related to the transformed data, i.e. a log-scale. Because percentage effects on a log-scale are difficult to interpret, we suggest back-transforming the MDD to the abundance scale and using this MDD for evaluation.

If the transformation *y*(*x*) = ln(ax + 1) is used as suggested by Van den Brink et al. (2000), the MDD for the abundance (MDD_abu_) can be calculated from the MDD given for the transformed data (MDD_ln_) with the following formula, using the back-transformation, *x* = (exp(*y*(*x*)) − 1) / *a*, and the arithmetic mean of the transformed control values, mean_co,ln_:

4 $$ MD{D}_{abu} = \left( \exp \left( mea{n}_{co, \ln}\right)-1\right)/ a\ \hbox{--}\ \left( \exp \left( mea{n}_{co, \ln}\hbox{--}\  MD{D}_{\ln}\right)-1\right)/ a $$

which can be simplified to

5 $$ MD{D}_{abu} = \left( \exp \left( mea{n}_{co, \ln}\right)\ \hbox{--}\ \exp \left( mea{n}_{co, \ln}\hbox{--}\ MD{D}_{\ln}\right)\right)/ a $$

The %MDD_abu_ is the MDD_abu_ related to the back-transformed mean of the controls.

6 $$ \% MD{D}_{abu} = 100\  MD{D}_{abu}/\left(\left( \exp \left( mea{n}_{co, \ln}\right)-1\right)/ a\ \right) $$

Here, the back-transformed mean of the controls corresponds to the geometric mean of the controls.

An example calculation of the MDD_ln_ and MDD_abu_ for abundance data analysed using the Williams’ test is given in the Supplementary Information, section A (SI A).

## How to reduce the MDD of microcosm/mesocosm experiments

### Factors affecting the MDD

**Fig. 1:**
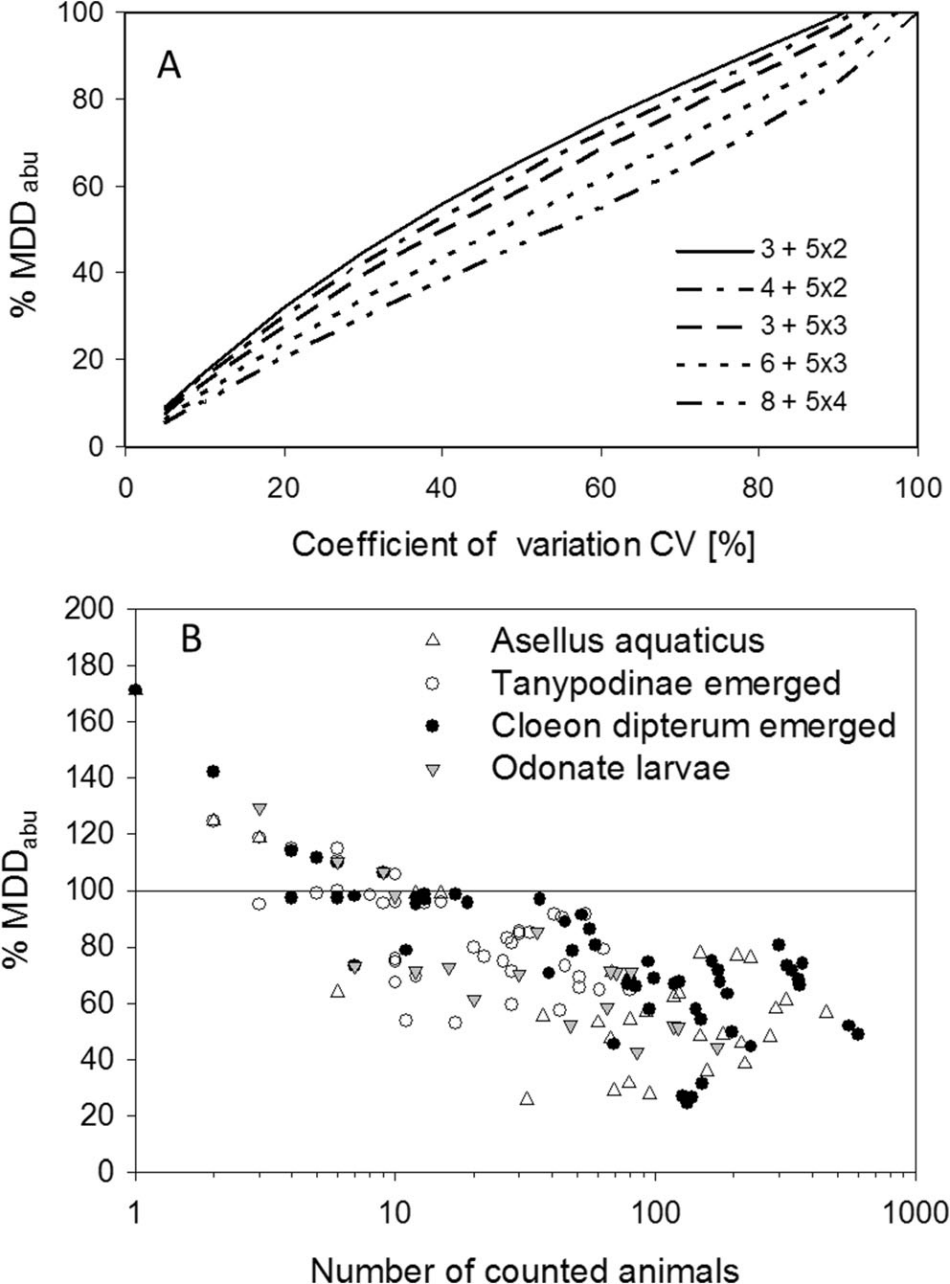
**a** Relationship between the coefficient of variation of a given endpoint and the MDD_abu_ for log-transformed (ln(2x + 1)) abundance data as influenced by different numbers of replicates in both controls and treatments. Scenario—one-sided Williams’ test (*p* = 0.05), MDD_abu_ shown for the fifth treatment level, mean abundance in control = 10. Explanation legend—3 + 5x2 = 3 control replicates and five treatment levels with two replicates. **b** Example of the relation between abundance (sum of individuals counted in four control ponds) and the MDD_abu_ for four groups of macro-invertebrates in an outdoor mesocosm study (one-sided *t* test (*p* = 0.05)), transformed data (log factor *a* = 2); data based on four control ponds. Assumption—four controls and three treatment replicates with the same coefficient of variation
  The variance caused by the differences between replicates should be minimised when constructing and preparing the test systems and by measures taken during the pre-experimental period (e.g. by means of mixing techniques to evenly distribute water and organisms over test systems). The sampling error can be reduced by increasing the number of individuals sampled and/or counted, and thus the MDD_abu_ very often decreases with increasing numbers of counted individuals, mainly due to the reduction of variability between samples (Fig. 1b). Hence, MDD_abu_s can be reduced by improving sampling techniques that increase the number of individuals sampled and scored per test system. The data presented in Fig. 1b suggest that for the evaluated species, at least 10–40 individuals need to be sampled to obtain a relatively low %MDD_abu_, above which there seems to be a point of diminishing returns for increasing the number of individuals sampled.
  For most organism groups, it is possible to improve the sampling efficiency: A reduction of the MDD_abu_ in the range of 5–25 % or more may be achieved by doubling either the sampling volume (e.g. for zooplankton) or the number of sampling devices (e.g. two emergence traps per test unit for insects; see Fig. 1b and SI Table 3 in Supporting Information, section C). As a typical outcome of a microcosm/mesocosm data analysis, the MDD_abu_ values based on combined data of two sampling devices are usually lower than the MDDs of individual sampling devices. An example is given in SI Fig. 1 (Supporting Information, section C) for the insect *Chaoborus crystallinus* sampled from two emergence traps.
  The water volume collected to determine the zooplankton, as well as the number of subsamples evaluated in phytoplankton quantification, can be adapted with respect to the number of organisms in the sample. Improvements to the methods could also include habitat-specific sampling (e.g. emergence traps located above macrophytes instead of traps above the open water column for specific insect species) and the use of additional types of sampling devices with higher trapping rates for specific organisms. Increasing the number of individuals counted by such methods will significantly increase the statistical power by reducing the %MDD value.
  Besides the options of using more efficient sampling methods and more replicates, it is also possible to group low-abundance taxa in a constructive way, e.g. on the basis of their taxonomy (e.g. family or order level) in order to obtain taxa with higher number of counted individuals and thus lower sampling error. Note that the evaluation of treatment-related effects in microcosm/mesocosm experiments should preferably be performed on a sufficient number of representative and potentially sensitive biological populations of water organisms at the species and/or genus level, since the selected ecological identity of the specific protection goals for aquatic algae, macrophytes and invertebrates is the population (see e.g. EFSA 2013; Nienstedt et al. 2012). The aggregation of taxa at a higher taxonomic level would reduce the level of taxonomic resolution and possibly also result in a grouping of sensitive and non-sensitive species and should therefore be done only if the MDDs of the non-aggregated taxa are too high for evaluation.
  It needs mentioning that in designing outdoor microcosm/mesocosm tests, it is impossible to know a priori which species will be present in appropriate densities due to unpredictable outdoor environmental conditions (e.g. weather) and stochastic events. Nevertheless, it is possible to design microcosm/mesocosm experiments in such a way that it is likely that a sufficient number of species representative for the taxonomic groups at risk will be present (e.g. arthropod species when studying insecticides and algae and macrophyte species when studying herbicides).
  In conclusion, more replicates will increase the statistical power. However, in the context of realistic scenarios for outdoor mesocosm studies, even an increase from two to four treatment replicates will reduce the MDD_abu_ only by a maximum of 11 % (at 60 % CV). By contrast, improving the sampling and quantification methods will often be of greater benefit with respect to reducing the MDD_abu_ values without the need to increase the replicate number. However, while increasing the sampling efficiency, one should avoid significantly depleting the populations just by sampling.

Equation 2 suggests that the MDD is affected by three factors:

1. The number of replicates *n*_0_, *n*
  Increasing the number of replicates reduces the square root term in Eq. 2, but it also increases the degrees of freedom of the test and thus the critical *t*-value.
2. The variance *s* ^2^
  The MDD is directly proportional to the variance of the measurement endpoints, which can be separated into the inherent variability between the replicates and the variability caused by the sampling methods (sampling error)
3. The selected error level *α*
  As the critical *t*-value also depends on the error level *α*, the decision on *α* also affects the MDD. However, we will keep the default error level of 0.05 here.
  The current Aquatic Guidance Document (EFSA 2013) recommends five or more test concentrations with at least two, but preferably more replicates per treatment level. In addition, it is advised to have a higher number of replicates for the control than that used for each treatment. For practical reasons, the total number of test systems in a microcosm/mesocosm study is often below 20 and usually below 30. Thus, we will focus here on designs with five test concentrations and different numbers of replicates.
  Figure 1a shows the %MDD_abu_ in relation to the coefficient of variation in the data set for different experimental designs using a ln (2x + 1) transformation, which is characteristic of macro-invertebrate data sets. It is obvious that the variation in the data has a stronger effect on the MDD_abu_ than just increasing the number of replicates. Nevertheless, for a given coefficient of variation (CV = standard deviation / mean), the increase in the number of control and treatment replicates clearly reduces the MDD_abu_ of a specific measurement endpoint. For example, increasing the number of treatment replicates from two to three (always using three control replicates) yields a %MDD_abu_ reduction in the range of 2–7 %, depending on the assumed CV. Increasing only the number of control replicates, e.g. from three to six controls (always using three treatment replicates), also results in an increase in statistical power (up to 7 % reduction in this case). Considering the practical limitations of increasing the number of test units (e.g. costs in constructing and managing replicate test systems; manpower for sampling, identification and counting of sampled organisms), it seems useful to reduce other sources of variation, e.g. the sampling error. For further background information on the influence of the number of replicates on the MDD, see the Supporting Information, section B (SI-B) and SI Table 2.

## How to report the MDDs for endpoints derived from microcosm/mesocosm tests

The MDDs should be reported together with the NOEC table for each taxon and time point. In order to allow the analysis to be reproduced, we suggest presenting the raw data (abundance per taxon, day and test unit) as well as tables with means of the transformed data, the re-transformation of the means and the two MDDs related either to the transformed or abundance data in an appendix. An example of the latter is given in SI Table 4 (in Supporting Information, section D).

If, for a specific taxon on a specific sampling day, the MDD_abu_ is <100 %, a treatment-related decline in abundance can in theory be demonstrated. If the MDD_abu_ is >100 %, however, the power of the test is too low to demonstrate treatment-related declines in abundance. Note, however, that in some cases of treatment-related increases (due to indirect effects), a statistically significant effect may be demonstrated if the MDD_abu_ is >100 %. Since RACs for pesticides derived from microcosm/mesocosm tests are in the vast majority of cases based on treatment-related declines in the abundance of sensitive populations, we will focus on the significance of MDD_abu_ values for the interpretation of treatment-related declines.

Following the Aquatic Guidance Document (EFSA 2013), the %MDD values can be clustered into five classes (Table 1). These MDD classes can be used to categorise taxa sampled in the microcosm/mesocosm experiment on the basis of their MDDs.

**Table 1:** Classes of minimum detectable differences (MDD) as proposed in the EFSA Aquatic Guidance Document

MDD class	%MDD	Comment
0	>100 %	No effects can be determined statistically
I	90–100 %	Only large effects can be determined statistically
II	70–90 %	Large to medium effects can be determined statistically
III	50–70 %	Medium effects can be determined statistically
IV	<50 %	Small effects can be determined statistically

Source: EFSA 2013)

Note that these classes apply to treatment-related reductions in abundance/biomass of taxa in particular, since the MDD may be larger than 100 % while treatment-related increases in abundance/biomass may still be demonstrated. We have assumed that the MDD in the EFSA Aquatic Guidance document would equal the MDD_abu_ as defined in this manuscript

In the present paper, we distinguish three categories of taxa on the basis of their MDD_abu_:

1. Taxa characterised by a sufficient statistical power to potentially demonstrate treatment-related responses and consequently also a no adverse effect concentration. For this, we propose the following MDD criterion using the MDD classes in Table 1:
  After the first application of the test item, the MDD_abu_ is
  - <100 % at no less than five samplings, or
  - <90 % at no less than four samplings, or
  - <70 % at no less than three samplings, or
  - <50 % at no less than two samplings.
  Species 1 and 2 in SI Table 4 (see Supporting Information, section D) fall into this category. Other examples of category 1 taxa are presented in the section “Examples to illustrate decision scheme 2 for treatment-related declines” of this paper. Note that this category is relevant to all taxa that show consistent treatment-related declines in population abundance but may also include taxa characterised by statistically significant treatment-related increases.
2. Taxa that do not meet the MDD_abu_ criterion that is mentioned under bullet point 1, but for which a LOEC can be calculated on at least one sampling. This category comprises taxa that are characterised by statistically significant decreases in population abundance on samplings when the MDD_abu_ values are <100 % (e.g. species 3 in SI Table 4). In addition, this category may comprise taxa characterised by statistically significant increases in population abundance on samplings for which (i) MDD_abu_ values are less than 100 % but which do not meet the conditions for category 1 taxa, (ii) MDD_abu_ values are higher than 100 % and (iii) MDD_abu_ values cannot be determined due to the absence of the taxon in controls. Examples of category 2 taxa characterised by treatment-related increases are presented in the section “Examples of effect class derivation in the case of treatment-related increases in population abundance” of this paper.
3. Taxa that do not meet the MDD_abu_ criterion mentioned under bullet point 1 and for which no significant difference with controls was found on any of the samplings (e.g. species 4 in SI Table 4).
  The statistical findings for each taxon belonging to a specific organism group and characterised by the same sampling methods (e.g. phytoplankton, zooplankton, macro-invertebrates and insect emergence) are used to construct summary tables on the basis of the above categories. These summary tables include, for each taxon (individual and grouped populations), the NOECs and the related MDD_abu_ for each sampling date. Category 1 taxa can be used to evaluate the reliability of a microcosm/mesocosm study to demonstrate treatment-related effects. Categories 1 and 2 taxa can be used for the effect classification of treatment-related effects. Category 3 taxa cannot be used in the evaluation of treatment-related responses and the derivation of effect classes (see sections below). An example of such a summary table is given in SI Table 5 of Supporting Information, section D.

## How to evaluate microcosm/mesocosm tests using MDDs and effect classes

### Reliability of a microcosm/mesocosm test for RAC derivation

**Fig. 2:**
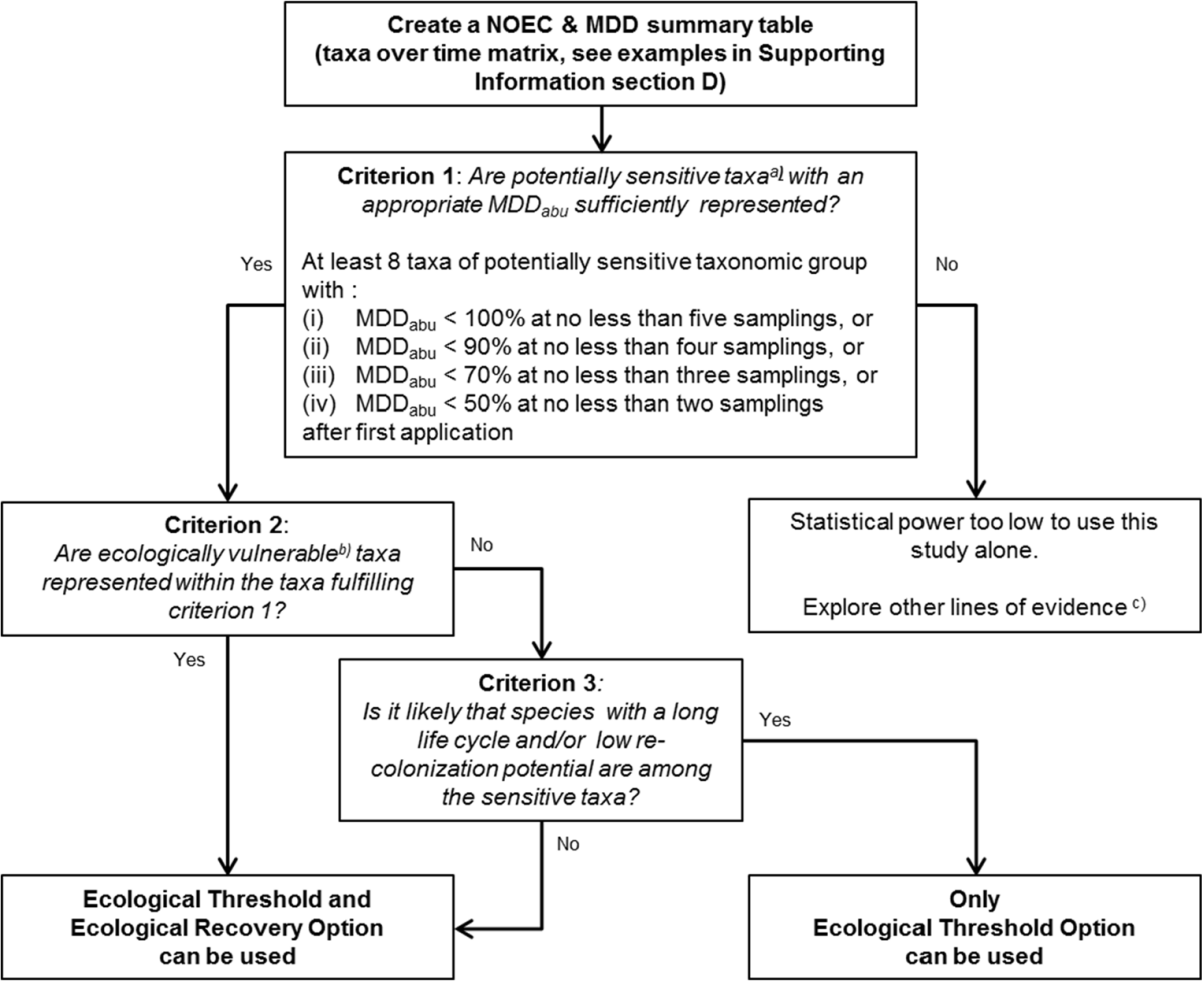
Decision scheme 1 to assess the reliability of a microcosm/mesocosm study to derive regulatory acceptable concentrations (RACs) on the basis of treatment-related effects of pesticide exposure. (^a)^)Informed by e.g. available single species and semi-field tests and other read-across information. (^b)^)Ecologically vulnerable due to potential intrinsic sensitivity to the test item, likelihood of exposure, long life cycle (e.g. bi-, uni- or semi-voltine) and/or low immigration potential. (^c)^)For example, focussed population-level and microcosm/mesocosm studies addressing additional sensitive species or population modelling

The reliability of a microcosm/mesocosm study to derive a higher tier RAC in the registration procedure for pesticides can be assessed by means of decision scheme 1 (Fig. 2). This decision scheme addresses three important criteria that, according to the EFSA Aquatic Guidance Document (EFSA 2013), need to be fulfilled to derive an RAC based on the ecological threshold option (ETO-RAC) and/or RAC based on the ecological recovery option (ERO-RAC).

Criterion 1 refers to the requirement that at least eight populations of potentially sensitive taxa with an appropriate MDD_abu_ should be present in the test systems, in the sense that the power of the test for these taxa is high enough to demonstrate possible treatment-related responses in terms of abundance (category 1 taxa). Potentially sensitive taxa are identified based on the toxic mode-of-action of the test item and on available ecotoxicological data (single-species toxicity tests; other semi-field experiments and/or read-across information for other pesticides with a similar mode-of-action). According to the EFSA Aquatic Guidance Document (EFSA 2013), representatives of primary producers (algae and macrophytes) can be considered the potentially sensitive taxa for herbicides and arthropods (insects and crustaceans) for insecticides. For fungicides with biocidal properties, the potentially sensitive taxa may be more diverse and comprise representatives of different taxonomic groups (e.g. algae, arthropods, worms). If, however, lower-tier and read-across data are available indicating that certain (standard) test species of primary producers (e.g. macrophytes for an herbicide), arthropods (e.g. insects for an insecticide) or water organisms in general (e.g. algae for a fungicide) are more than an order of magnitude more sensitive than the other test species, criterion 1 refers to representatives of such a sensitive taxonomic subgroup. For further guidance, we refer to the EFSA Aquatic Guidance Document (EFSA 2013). In cases where, based on lower tier toxicity data and read-across information, it is not fully known a priori what the potentially sensitive taxa are, some flexibility in the application of criterion 1 may be needed.

Criteria 2 and 3 in decision scheme 1 (Fig. 2) refer to the presence of ecologically vulnerable taxa amongst the populations of the potentially sensitive taxa with an appropriate MDD_abu_ (criterion 1). Properties relevant to defining the vulnerability of non-target organisms to pesticides are species traits that determine (i) susceptibility to exposure (e.g. relating to habitat preference and the ability to avoid exposure) and (ii) toxicological sensitivity (e.g. relating to the specific toxic mode-of-action of the pesticide and the properties of the organisms to cope with pesticide uptake, and elimination and repair of damage) and internal and external recovery processes (e.g. relating to generation time, number of offspring, dispersal ability and connectivity to nearby refugia) (Caquet et al. 2007; Brock et al. 2010; De Lange et al. 2010; Kattwinkel et al. 2012; Rubach et al. 2012). If several representative vulnerable populations are present (criterion 2) among the potentially sensitive taxa fulfilling criterion 1 and it is likely that species with a long generation time and/or low recolonisation potential are amongst the sensitive taxa (criterion 3), the study may be used to derive RACs on the basis of both the ETO-RAC and the ERO-RAC. With respect to criterion 3, it is important to note that certain vulnerable taxa may occur in specific habitats only (e.g. Plecoptera in lotic waters or floating macrophytes in lentic waters). Furthermore, species such as gammarids that often show a high recolonisation potential in interconnected field habitats (e.g. streams and ditches) may have a low recolonisation potential in isolated microcosm/mesocosm. If potentially vulnerable taxa are not sufficiently represented in the microcosm/mesocosm test systems, the concentration–response relationships for the potentially sensitive taxa can only be used to derive an ETO-RAC (for further guidance, see EFSA 2013).

### Effect classification and ETO-RAC and ERO-RAC derivation

The next step is the evaluation of the microcosm/mesocosm study on the basis of effect classes incorporating the MDD concept. For this, we propose to slightly adapt the effect classes presented by De Jong et al. (2008) and in the EFSA Aquatic Guidance Document (EFSA 2013), so as to better integrate the MDD requirements

**Fig. 3:**
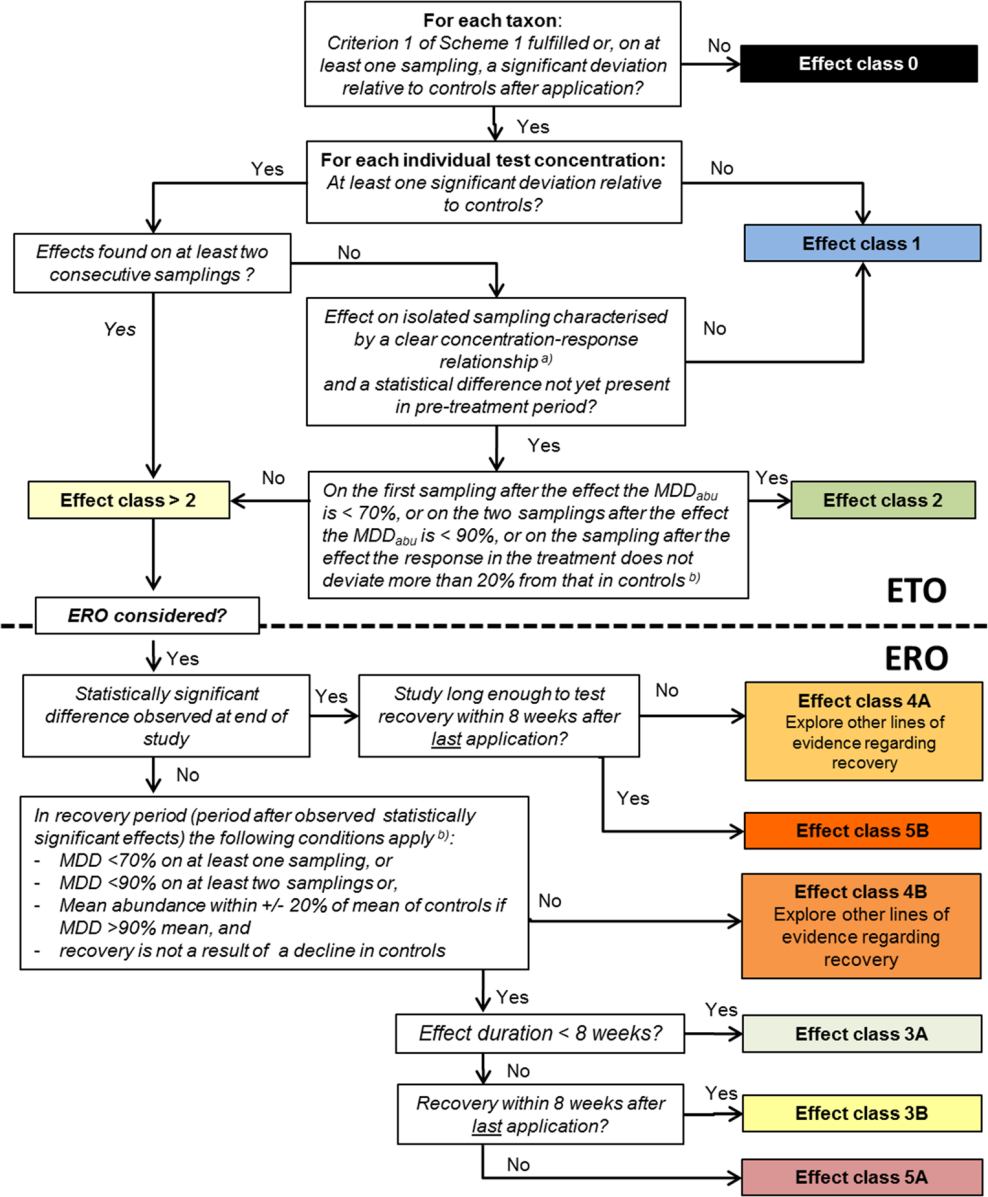
Decision scheme 2 for the derivation of effect classes for treatment-related effects (focus on treatment-related declines) on population abundance from results of microcosm/mesocosm studies. The MDD_abu_ values mentioned in the decision scheme are not applicable to indirect effects in the form of increases in population abundance if the NOECs of these treatment-related increases are associated with MDD_abu_ values >100 % or if no MDD_abu_ can be calculated due to the absence of the taxon in control test systems (n.c.). (^a)^) A clear concentration–response relationship for direct effects is characterised by a monotonous treatment-related decrease in abundance while in addition, the statistical difference coincides with a high enough mean abundance of the taxon in controls. When selecting a certain minimum abundance for a taxon in controls, the argumentation for this should be provided. If the significant effect is observed in the application period, the next sampling should occur within a week. (^b)^) If the high %MDD_abu_ in the post-effect period can be explained ecologically (e.g. emergence of insects) and a justification is given that this phenomenon will also occur under realistic field conditions, some flexibility of the MDD criterion is recommended
Effect class 3A (Pronounced short-term effects (effect period <8 weeks), followed by recovery)
  Clear response of sensitive endpoints, but full recovery of affected endpoints within 8 weeks after the first application or, in case of delayed responses and/or repeated applications, the duration of the effect period is less than 8 weeks and is followed by full recovery. Treatment-related effects are demonstrated on consecutive samplings. Note that according to decision scheme 2 (Fig. 3), recovery from treatment-related declines in abundance can only be considered if the MDD_abu_ values during the relevant recovery period are <70 % on at least one sampling, or <90 % on at least two samplings, or if the % deviation from controls is less than 20 %. If this is not the case, effect class 3B or 4B has to be selected.
Effect class 3B (Pronounced effects that last longer than 8 weeks but recovery observed within 8 weeks after the last application)
  Clear response of the endpoint in the microcosm/mesocosm experiment repeatedly treated with the test substance, lasting longer than 8 weeks (responses may already start in the treatment period), but full recovery of affected endpoints within 8 weeks post the last application. Note that according to decision scheme 2 (Fig. 3), recovery from treatment-related declines in abundance can only be considered if the MDD_abu_ values during the relevant recovery period are <70 % on at least one sampling and <90 % on at least two samplings, or if the % deviation from controls is less than 20 %. If this is not the case, effect class 4B has to be selected.
Effect class 4A (Significant effects in short-term study)
  Clear effects (e.g. large reductions in densities of sensitive species) observed, but the study was too short to demonstrate complete recovery within 8 weeks after the (last) application. This effect class is also applicable in case of delayed responses observed at the end of the study. If a delayed response is observed on the last sampling only, this may be indicated as effect class 2–4A. If the delayed response is demonstrated for several consecutive samplings at the end of the study and the demonstrated effect period is <8 weeks, this may be indicated as effect class 3A–4A. Other lines of evidence may be provided to re-address effect class 4A. These other lines of evidence may comprise focussed indoor toxicity tests, outdoor population-level tests and/or mechanistic modelling approaches with the taxon of concern.
Effect class 4B (Significant short-term effects demonstrated but recovery cannot be properly evaluated due to high %MDD_abu_ values in recovery period)
  Clear effects (e.g. large reductions in densities of sensitive species) observed, statistically significant differences from controls last less than 8 weeks but recovery cannot be evaluated, e.g. due to MDD_abu_ values >100 % or due to pronounced population decline in controls in the recovery period after a treatment-related decline. If a significant treatment-related response is demonstrated on one sampling but recovery cannot be interpreted due high MDDs, we suggest to indicate this with an effect class 2–4B. If the responses are demonstrated for several consecutive samplings, we suggest indicating this with an effect class 3A–4B. Other lines of evidence may be provided to re-address effect class 4B. These other lines of evidence may comprise focussed indoor toxicity tests, outdoor population-level tests and/or mechanistic modelling approaches with the taxon of concern.
Effect class 5A (Pronounced long-term effect followed by recovery)
  Clear response of sensitive endpoint, effect period longer than 8 weeks and recovery does not yet occur within 8 weeks after the last application, but full recovery is demonstrated to occur in the year of application. Note that according to decision scheme 2 (Fig. 3), recovery from treatment-related declines in abundance can only be considered if the MDD_abu_ values during the relevant recovery period are <70 % on at least one sampling and <90 % on at least two samplings or if the % deviation from controls is less than 20 %. If this is not the case, effect class 5B has to be selected.
Effect class 5B (Pronounced long-term effects without recovery)
  Clear response of sensitive endpoints (>8 weeks post the last application) and full recovery cannot be demonstrated before termination of the experiment or before the start of the winter period.

Effect class 0 (Treatment-related effects cannot be evaluated statistically. If this class is consistently assigned to endpoints/taxa that are deemed most relevant for the interpretation of the study, the regulatory reliability of the microcosm/mesocosm tests is questionable)
  Effect class 0 is used for all category 3 taxa, while the effect classes mentioned below can be used for category 1 and category 2 taxa.
Effect class 1 (No treatment-related effects demonstrated; NOEC_population_)
  No (statistically and/or ecologically significant) effects observed as a result of the treatment. Observed differences between treatment and controls show no clear causal relationship. Note that besides statistical support, a clear causal relationship also needs biological support (e.g. based on ecotoxicological lower tier information and the ecology of the populations present in the test systems).
Effect class 2 (Slight effects)
  Statistically significant effects concern short-term and/or quantitatively restricted responses usually observed at individual samplings only. Note that according to decision scheme 2 (Fig. 3), recovery from the isolated treatment-related decline in abundance can only be considered if the MDD_abu_ value on the sampling after the effect is <70 % or if the value on the two samplings after the effect is <90 %, or if on the sampling after the effect, the % deviation from controls is less than 20 %. If this is not the case, effect class 3A or 4B has to be selected.

### Effect classes and treatment-related responses in terms of abundance

In order to use a reliable microcosm/mesocosm experiment for RAC derivation, an important task is to derive an effect class for each taxon and each concentration (i.e. treatment level). Considering MDD_abu_ values when deriving effect classes is important for answering two different questions, viz., (1) can we reliably demonstrate a NOEC and (2) can we state that a population has recovered after a period of statistically significant effects? From a statistical point of view, it is only possible to prove an effect. So, the demonstration of no treatment-related effects underlying both questions relies on the statistical power to detect an effect, and the MDD_abu_ is the proxy for this statistical power. Within this context, two aspects are of importance. First, when a statistically significant effect can be demonstrated, the MDD_abu_ does not hamper the detection of an effect and the calculation of a NOEC/LOEC. Second, when no statistically significant effect can be demonstrated, the MDD_abu_ becomes important to show the ability to detect the treatment-related decline in population abundance and to calculate a corresponding NOEC/LOEC. To demonstrate treatment-related decreases, the MDD_abu_ should at least be <100 %, but to detect statistically significant increases in population abundance, the MDD_abu_ values may be either smaller or larger than 100 %. Figure 3 presents a flowchart (decision scheme 2) to derive effect classes for treatment-related declines in abundance (see also examples in the section Examples to illustrate decision scheme 2 for treatment-related declines). How to assign effect classes to treatment-related increases in population abundance is discussed below on the basis of some example case studies presented in the section Examples of Effect class derivation in the case of treatment-related increases in population abundance.

Decision scheme 2 (Fig. 3) firstly assesses the potential of effect class 1 and 2 derivation underlying the ETO-RAC and secondly searches for the potential for effect class 3A derivation underlying the ERO-RAC (following the proposal in EFSA 2013).

In the first step, all category 3 taxa (not fulfilling MDD_abu_ criterion 1 and showing no statistically significant effects) which were present in the study under evaluation are excluded from the analysis and allocated to effect class 0. This just means that it was not possible to decide if there was an effect or not. If these species are of concern, other lines of evidence have to be evaluated.

In the second step, the taxa that fulfil MDD_abu_ criterion 1, but for which no effects could be demonstrated, are allocated to effect class 1. For these taxa, the statistical power was high enough and either no effects were found or only statistically significant differences with controls on an isolated sampling without a clear concentration–response relationship. The second distinction is important due to the high number of statistical tests which are conducted to evaluate a mesocosm study. Using an alpha of 0.05 assumes that five out of 100 tests will result in false positives. For example, by assuming 24 species on 8 sampling dates and applying the Williams’ test, we end up with 192 test results, 10 of which may be false positives just by chance.

If statistically significant effects with a clear concentration–response relationship are demonstrated on an isolated sampling and this effect is likely to be of limited magnitude (e.g. less than 50 %) and duration, while the statistical power is also high enough on the samplings after the statistically significant effect, effect class 2 is chosen.

If statistically significant effects are found on at least two consecutive samplings, effect classes higher than 2 have to be chosen. This also means that if the risk assessment is based on the ecological threshold option, the analysis can stop for this treatment level, because the higher effect classes cannot be used to derive an ETO-RAC. If recovery after an effect period of at least two samplings is assessed, the question at stake is if we can demonstrate that the statistical power of the study was high enough to define recovery of that taxon. Consequently, the MDD_abu_ values in the recovery period become important.

In the third step, all species/taxa are selected for which no recovery could be demonstrated, and either effect class 4A or 5B is selected, based on the study design. Selecting effect class 5B indicates that the species/taxon was unable to recover in the study under evaluation. Selecting effect class 4A means that it cannot be excluded that the taxon may recover after an effect period <8 weeks, but that this cannot be demonstrated in the study, so that other lines of evidence have to be evaluated. These other lines of evidence may comprise focussed indoor toxicity tests and outdoor population-level experiments, particularly in combination with mechanistic modelling approaches with the taxon of concern (e.g. Preuss et al. 2009; Galic et al. 2010; Gabsi et al. 2014; Baveco et al. 2014). If a delayed treatment-related response is observed at the end of the study, an effect class 2–4A (delayed effect on last sampling) or 3A–4A (delayed effects on consecutive samplings at the end of the study) may be selected to better summarise the treatment-related response information.

In the fourth step, the species/taxa are addressed for which recovery could not be demonstrated because of low statistical power in the post-effect period (or in the case of an MDD_abu_ >90 %, the deviation of means in the treatment was larger than 20 % when compared with controls); for these species, effect class 4B is selected. This does not mean that the species does not have the potential to recover but that it was not possible to demonstrate this in the study under evaluation, and other lines of evidence (e.g. additional experimental and population modelling approaches) are necessary to demonstrate recovery for this taxon at this concentration (treatment level), if necessary. Depending on the number of samplings, a statistically significant response was demonstrated in which an effect class 2–4B or 3A–4B may be selected to better summarise the treatment-related response information.

In the last step, for all the species for which an effect and recovery could be demonstrated in the study, the time of recovery becomes important in order to select effect class 3A, effect class 3B or effect class 5A. Note that in deriving an ERO-RAC, only effect class 3A effects are considered acceptable according to the current EFSA Aquatic Guidance Document (EFSA 2013).

## Examples to illustrate decision scheme 2 for treatment-related declines

**Fig. 4:**
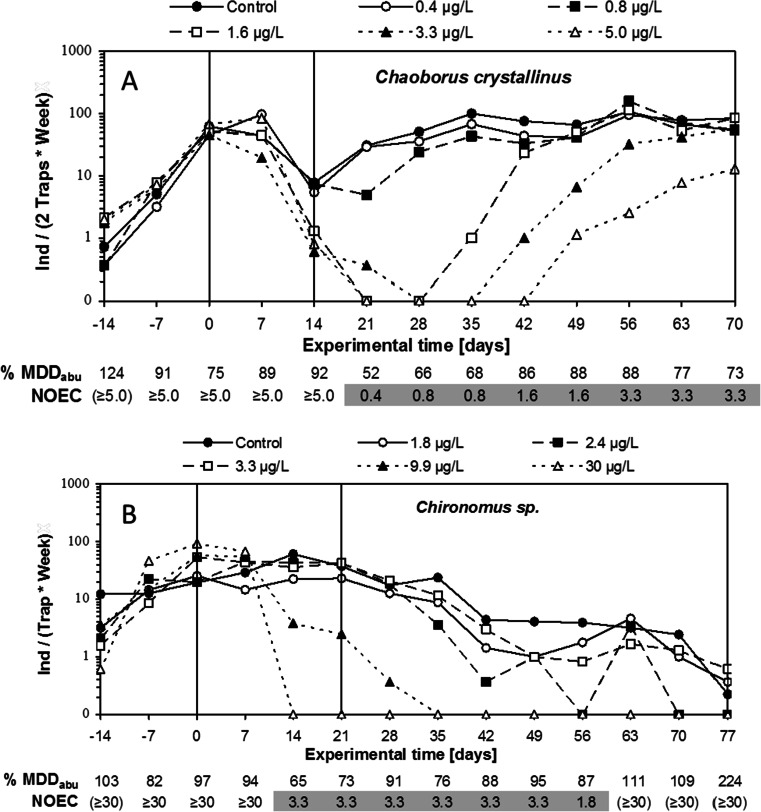
**a** Dynamics of the numbers of adult *Chaoborus crystallinus* collected in emergence traps placed in mesocosms treated twice (days 0 and 14) with different concentrations (0–5.0 μg/L) of an insecticide. **b** Dynamics of the numbers of adult *Chironomus* sp. collected in emergence traps placed in mesocosms treated twice (days 0 and 21) with different concentrations (0–30 μg/L) of another insecticide. Shown *below each panel* are the calculated %MDD_abu_ and NOEC values for each sampling day. If a NOEC is placed between brackets, this means that the corresponding %MDD_abu_ value is >100 % and a proper NOEC for treatment-related decline cannot be derived

This section presents two examples (Fig. 4a, b) to illustrate how decision scheme 2 (Fig. 3) can be used to derive effect classes from typical treatment-related declines in abundance as observed in microcosm/mesocosm tests.

Figure 4a presents the dynamics of the abundance of the phantom midge *C. crystallinus* as sampled in emergence traps placed in mesocosms that were treated twice (days 0 and 14) with an insecticide (concentrations 0.4–5.0 μg/L). In the post-treatment period, %MDD_abu_ values ranged between 52 and 92 %, and statistically significant declines in abundance were observed from day 21 up to and including day 63 (NOECs from 0.4–3.3 μg/L). In the mesocosms that received the lowest insecticide concentration (0.4 μg/L), no statistically significant effects were observed. In the 0.8 μg/L mesocosms, statistically significant declines in *C. crystallinus* were observed on day 21 only, followed by full recovery (note that the %MDD_abu_ level was 66 % on day 28). In the mesocosms that received 1.6 and 3.3 μg/L, statistically significant declines were observed in the periods days 21–35 and days 21–49, respectively, followed by full recovery (%MDD_abu_ values <90 % on all samplings in the recovery period). In the mesocosms that received the highest concentration (5 μg/L), statistically significant effects were observed from day 21 up until the last sampling day. Using decision scheme 2 (Fig. 3), the following effect classes can be derived for the treatment-related effects of the insecticide on *C. crystallinus*:

- Effect class 1, 0.4 μg/L
- Effect class 2, 0.8 μg/L
- Effect class 3A, 1.6–3.3 μg/L
- Effect class 5B, 5.0 μg/L

Figure 4b presents the dynamics of the abundance of *Chironomus* sp. as sampled in emergence traps placed in mesocosms that were treated twice (days 0 and 21) with an insecticide (concentrations 1.8–30 μg/L). At the end of the experiment, the numbers of *Chironomus* sp. adults collected in emergence traps gradually declined in the controls and lower treatment levels. Statistically significant treatment-related declines were observed from day 14 up to and including day 56 (NOECs 1.8–3.3 μg/L), and in this period, %MDD_abu_ values ranged between 65 and 95 %. After day 56, however, %MDD_abu_ values were larger than 100 %, so recovery in the two highest treatments (9.9 and 30 μg/L) could not be assessed. On day 87, an isolated NOEC of 1.8 was calculated. No consistent concentration–response relationship could be demonstrated on this sampling day, however, so that this isolated NOEC should be interpreted with caution and can probably be considered an example of a false positive. Using decision scheme 2 (Fig. 3), the following effect classes can be derived for the treatment-related effects of the insecticide on *Chironomus* sp.:

- Effect class 1, 1.8–3.3 μg/L
- Effect class 3A–4B, 9.9–30 μg/L

## Examples of effect class derivation in the case of treatment-related increases in population abundance

**Fig. 5:**
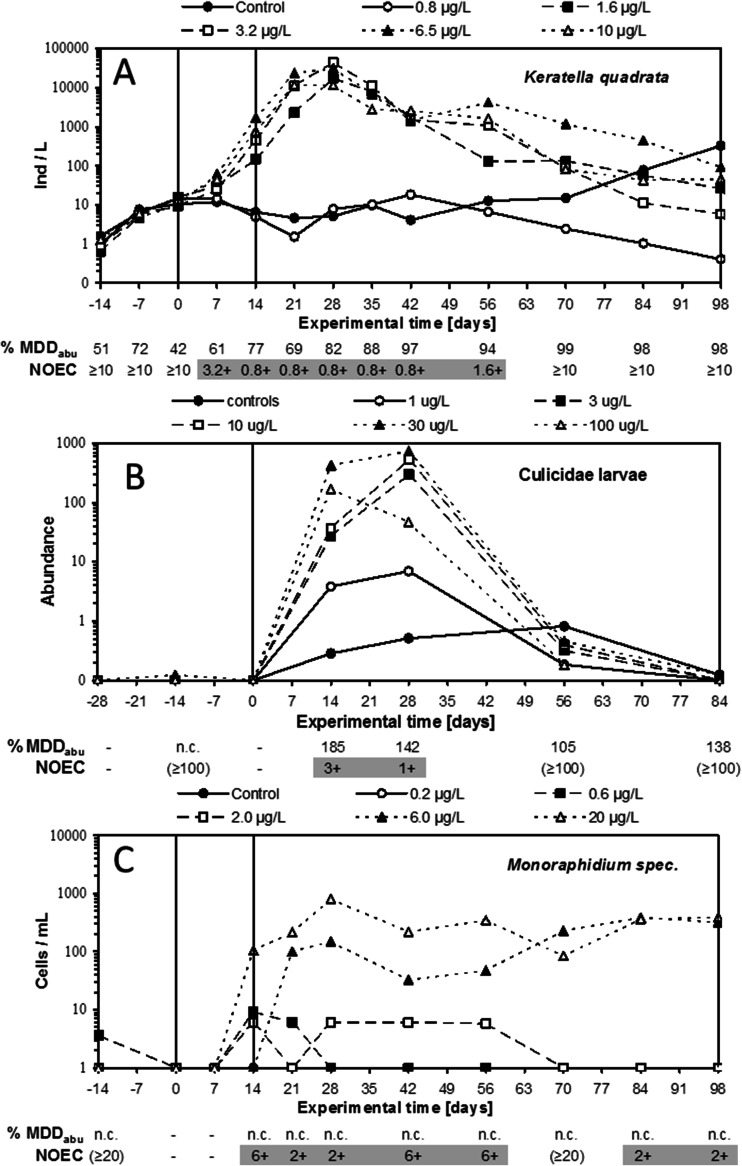
**a** Dynamics of the numbers of the rotifer *Keratella quadrata* in zooplankton samples of a mesocosm treated twice (days 0 and 14) with different concentrations (0.8–10 μg/L) of an insecticide. **b** Dynamics of the numbers of Culicidae midge larvae in net samples from a mesocosm treated once (1–100 μg/L) with a fungicide. **c** Dynamics of the abundance of the green alga *Monoraphidium* in phytoplankton samples from a mesocosm treated twice (days 0 and 14) with different concentrations (0.2–20 μg/L) of an insecticide. Shown *below each panel* are the calculated %MDD_abu_ and NOEC values for each sampling day (a ‘+’ behind NOEC value indicates a treatment-related increase). If a NOEC is placed between brackets, this means that the corresponding %MDD_abu_ value is >100 % and a proper NOEC for treatment-related decline cannot be derived

This section presents three examples (Fig. 5a–c) to illustrate how to derive effect classes for typical treatment-related increases in abundance as observed in microcosm/mesocosm tests.

In Fig. 5a, the rotifer species *Keratella quadrata* shows a consistent treatment-related increase in abundance in the period from day 0 up to and including day 56, and the %MDD_abu_ values are <100 % on all samplings, indicating that relatively small treatment-related increases can be determined statistically. The lowest NOEC calculated is 0.8 μg/L on five consecutive samplings, resulting in statistically significant increases in *Keratella* for a period of 32–49 days in the mesocosms treated with 1.6 μg/L. In the test systems that received 3.2 μg/L, statistically significant increases in abundance were observed from day 14 up to and including day 94 (effect period 56–73 days but recovery observed within 56 days post the last application). In the test systems that received the two highest concentrations (6.5 and 10 μg/L), statistically significant increases were observed for 63–70 days, but effects could no longer be demonstrated 56 days post the last application. Recovery from the treatment-related increase is apparent at the end of the experiment as shown by an increase in the abundance of *Keratella* in the controls. On the last sampling, the mean abundance values in the controls were even higher than in the insecticide-treated systems. Note, however, that at the last three samplings, the concentration–response relationship becomes less linear. This may be caused by stochastic processes that cause replicate test systems to deviate as time proceeds.

Using the effect classification presented in the section Effect classification and ETO-RAC and ERO-RAC derivation, the following effect classes can be derived for the treatment-related effects of the insecticide on *K. quadrata*:

- Effect class 1, 0.8 μg/L
- Effect class 3A, 1.6 μg/L (indicative of an increase)
- Effect class 3B, 3.2–10 μg/L (indicative of an increase)

In Fig. 5b, Culicidae midge larvae show a statistically significant treatment-related increase on sampling days 14 and 28. On these dates, the %MDD_abu_ values were 185 and 142 %, respectively. These %MDD_abu_ values >100 % indicate that the experimental design of the study allows the detection of treatment-related increases (relative to controls) of medium size only. On day 28, a statistically significant increase was observed in the test systems that received 3 μg/L and higher, while on day 14, this was observed for the 10–100 μg/L treatment levels. On day 56 (after the single application), full recovery from the treatment-related increase was observed, since the mean abundance values of all treated systems on this sampling day were below the mean control value. Although in the 3 μg/L test systems, statistically significant effects were observed on one sampling only, and effect class 3A is assigned to this treatment level because of the relatively pronounced effect observed and the wide sampling intervals of 14–28 days.

Using the effect classification presented in the section Effect classification and ETO-RAC and ERO-RAC derivation, the following effect classes can be derived for the treatment-related effects of the fungicide on Culicidae:

- Effect class 1, 0.1 μg/L
- Effect class 3A, 3–100 μg/L (indicative of an increase)

In Fig. 5c, the green alga *Monoraphidium* sp. shows a statistically significant treatment-related increase on several consecutive samplings in the tests systems that received 6.0 and 20 μg/L. Note that for this species, no %MDD_abu_ could be calculated for samplings at which the taxon was not observed in any of the test systems (indicated by the symbol ‘-’ on days 0 and 7) or did not occur in the controls (indicated by symbol ‘n.c.’ on all other sampling days). Nevertheless, a NOEC for a treatment-related increase of 2 μg/L could be calculated on sampling days 21, 28, 84 and 96 so also at the end of the experiment.

Using the effect classification presented in the section Effect classification and ETO-RAC and ERO-RAC derivation, the following effect classes can be derived for the treatment-related effects of the insecticide on *Monoraphidium* sp.:

- Effect class 1, 0.2–2.0 μg/L
- Effect class 5B, 3–100 μg/L (indicative of an increase)

## Concluding remarks

The first mesocosm experiments that evaluated the effects of pesticides on aquatic ecosystems were performed in the 1970s and the early 1980s. These experiments were done in very large systems which allowed only a limited level of experimental control and often included fish. As a result, these mesocosm experiments yielded data with a high variation between the replicates (e.g. Shaw et al. 1995). The statistical power was often investigated and seen as rather low to detect effects (Kraufvelin 1998). In order to reduce the variability between replicates, a trend was initiated in the 1990s to use smaller test systems which allowed a higher level of control and to exclude large predators like fish. The design of microcosm/mesocosm was also more fully aligned with the endpoints of interest, e.g. using small systems when plankton is the endpoint of interest and using larger outdoor systems when recovery of the insect community is of interest (Campbell et al. 1999). These changes to the experimental design of microcosm/mesocosm experiments probably greatly enhanced their statistical power, although no formal evaluation has ever been performed. The discussion about the statistical power of microcosm/mesocosm tests has received attention ever since and focussed on the number of replicates needed to detect a certain effect size (Sanderson 2002). In this paper, we show that the statistical power of microcosm/mesocosm experiments can also, or even to a larger extent, be increased by improving the sampling and quantification methods rather than by increasing the number of replicates alone.

In this paper, we also tried to formalise the use of the statistical power of microcosm/mesocosm experiments (expressed as the MDD) in their evaluation and the derivation of ecological threshold levels of no effect and acceptable effects. This protocolisation of the derivation of threshold values fulfils the request by EFSA (2013) for more practical experience in applying MDDs to evaluate results of microcosm/mesocosm experiments, which is required for the provision of more detailed guidance on MDD and the interpretation of microcosm/mesocosm endpoints. The recommendations presented in this paper may be used as input for the preparation of a specific view on the use of MDD and the evaluation of microcosm/mesocosm studies as specifically requested by EFSA’s PPR panel (EFSA 2013).

## Electronic supplementary material

Below is the link to the electronic supplementary material.

ESM 1 (DOCX 73 kb)

ESM 2 (XLSX 46 kb)
