# Supplementary material for: The minimum detectable difference (MDD) and the interpretation of treatment-related effects of pesticides in experimental ecosystems
Source: Environ Sci Pollut Res Int. 2014 Aug 15;22(2):1160–74. doi: 10.1007/s11356-014-3398-2 (PMC4544645; doi:10.1007/s11356-014-3398-2)
Supplement: Supplementary file 1 — (DOCX 73 kb) [file 11356_2014_3398_MOESM1_ESM.docx]

Supporting Information

##### **The minimum detectable difference (MDD) and the interpretation of treatment-related effects of pesticides in experimental ecosystems**

T.C.M. Brock, M. Hammers-Wirtz, U. Hommen, T. G. Preuss, H-T. Ratte, I. Roessink, T. Strauss, P.J. Van den Brink

**SI section A: Calculation examples for MDD_ln_ and MDD_abu_**

***SI Table 1****: Calculation example for Williams test including % MDD_ln_ and %MDD_abu_.*

| **Abundance data** |  |  |  |  |  |  |  |
| --- | --- | --- | --- | --- | --- | --- | --- |
| Replicate | Control | 0.1 mg/L | 0.3 mg/L | 1  mg/L | 3  mg/L | 10 mg/L |  |
| 1 | 175 | 29 | 27 | 36 | 26 | 20 |  |
| 2 | 65 | 114 | 78 | 11 | 13 | 37 |  |
| 3 | 154 | 72 | 27 | 105 | 33 |  |  |
| 4 | 83 |  |  |  |  |  |  |
|  |  |  |  |  |  |  |  |
| **Transformed data: y=ln(2*x)+1** | | |  |  |  |  |  |
| Replicate | Control | 0.1 mg/L | 0.3 mg/L | 1  mg/L | 3  mg/L | 10  mg/L | Sum |
| 1 | 5.86 | 4.08 | 4.01 | 4.29 | 3.97 | 3.71 |  |
| 2 | 4.88 | 5.43 | 5.06 | 3.14 | 3.30 | 4.32 |  |
| 3 | 5.73 | 4.98 | 4.01 | 5.35 | 4.20 |  |  |
| 4 | 5.12 |  |  |  |  |  |  |
| n | 4 | 3 | 3 | 3 | 3 | 2 | 18.00 |
| mean | 5.40 | 4.83 | 4.36 | 4.26 | 3.82 | 4.02 |  |
| sum | 21.59 | 14.49 | 13.07 | 12.78 | 11.47 | 8.03 |  |
| sum² | 117.18 | 70.92 | 57.68 | 56.88 | 44.31 | 32.43 | 379.40 |
| sum*sum/n | 116.50 | 69.97 | 56.95 | 54.42 | 43.86 | 32.25 | 373.95 |
| MLE | 5.40 | 4.83 | 4.36 | 4.26 | 3.92 | 3.92 |  |
| t |  | 1.103 | 2.020 | 2.210 | 2.870 | 2.531 |  |
| w |  | 1.33 | 1.33 | 1.33 | 1.33 | 2.00 |  |
| t(Williams 72, Tab. 1, 0.05, 1-sided) | | 1.782 | 1.873 | 1.903 | 1.918 | 1.927 |  |
| beta(Williams 72, Tab. 1) | | | 3.000 | 4.000 | 5.000 | 5.000 |  |
| t_critical |  | 1.782 | 1.866 | 1.893 | 1.906 | 1.902 |  |
| Significance |  | **-** | ***** | ***** | ***** | ***** |  |
| MDD ln |  | 0.92 | 0.96 | 0.97 | 0.98 | 1.11 |  |
| MDD% ln |  | 17.0 | 17.8 | 18.1 | 18.2 | 20.6 |  |
| MDD abu |  | 66.25 | 68.11 | 68.70 | 68.97 | 73.98 |  |
| MDD% abu |  | 60.3 | 62.0 | 62.5 | 62.8 | 67.3 |  |
|  |  |  |  |  |  |  |  |
|  |  |  |  |  |  |  |  |
| Total variance | 0.454 |  |  |  |  |  |  |
| d.f. | 12 |  |  |  |  |  |  |

See the supporting information provided as an Excel file.

**SI section B: Influence of the number of replicates on the MDD**

In the example presented in SI Table 2, increasing the number of replicates from 2 to 3 or 4 reduces the MDD by 26 and 36% (for the fifth out of five test concentrations), respectively. Furthermore, it is generally recommended to use more controls than treatment replicates (e.g. Williams 1972), often twice the number of replicates of the treatment levels. This reduces the MDD for the same example presented in SI Table 2 by 18, 15 and 15% for n = 2, 3 and 4, respectively. In addition to the statistical advantage, this offers the option of excluding control replicates in case of demonic intrusion or human errors that unintentionally affect a specific control test system in particular (EFSA 2013) without losing too much statistical power. Comparing the design with twice the number of controls only indicates a 24% improvement when switching from a 4 + 5x2 to a 6+5x3 design, while a further increase (8+5x4 design) has a smaller effect.

*SI Table 2: Effect of different experimental designs on the MDD according to Eq. 2. Calculations were done for the fifth of five test concentrations using critical t-values for the one-sided Williams test (Williams 1972). n_c = the replicate of the control, n_t = replicates per treatment level. For calculation see suppl. Info.)*

|  | n_t=2 | n_t=3 | n_t=4 | design |
| --- | --- | --- | --- | --- |
| increasing n, n_c = n_t |  | 26% | 38% | 6*2, 6*3 or 6*4 |
| doubling n_controls | 19% | 16% | 16% | e.g. 6+5*3 instead 4+5*3 |
| increasing n, n_c = 2 n_t |  | 24% | 36% | 6+5*3 or 8+5*4 instead 4 + 5*2 |
|  |  |  | 15% | 8+5*4 instead 6+5*3 |

Note that if data have to be transformed for the testing, the results presented in SI Table 2 hold only for the MDD of the transformed data (the MDD_ln_), while the effect on the MDD_abu_ depends on the type of the transformation and the mean abundance in the control.

**SI section C: Reducing the MDD_abu_ by reducing the variance of the data**

*SI Table 3: Example of the decrease in MDD_abu_ values due to doubling the number of emergence traps for different macroinvertebrate taxa from two outdoor mesocosm studies. Example: One-sided t-test (p=0.05), transformed data (log factor a = 2). The mean MDD_abu_ decrease was calculated as the mean value of all differences between the MDD_abu_ based on the sum of both traps and each of the two single-trap MDD_abu_ for every sampling date. Data based on 4 (study A) and 3 (study B) control ponds. Assumption: 4 and 3 controls, respectively, and 3 treatment replicates with the same variance.*

*SI Figure 1: Sum of emerged adults of* Chaoborus crystallinus *in 4 control ponds of the outdoor mesocosm study A (panel above), and the MDD_abu_ calculated for the combined traps and the two single traps (panel below). Example: one-sided t-test (p=0.05), transformed data (log factor a = 2); Data based on 4 control ponds. Assumption: 4 controls and 3 treatment replicates with the same variance.*

**SI section D: How to report the MDDs for endpoints derived from micro-/mesocosms**

*SI Table 4: Example of a table of the test results for all samplings per taxon: Range of controls and means of the ln-transformed data per treatment level including indications of significant difference with control (*), the % MDD_ln_ for the transformed data, values transformed back to the abundance scale and finally the NOEC and the MDD_abu_ related to the abundance data (one-sided Williams test, alpha = 0.05, MDD related to the NOEC).*

*SI Table 5: Example of a NOEC and % MDD_abu_ (in brackets) matrix for category 1, 2 and 3 taxa of a macro-invertebrate data set. n.c. indicates that the % MDD could not be calculated, e.g. because abundance was 0 in the controls. An ‘-’ indicates that a NOEC for a treatment-related decline could not be calculated due to an MDD>100%, while no significant treatment-related increase could be demonstrated either.
Note that the % MDD_abu_ is given in relation to the original abundance (not the transformed data used for the testing). ↓ = significant decrease in abundance/biomass; ↑ = significant increase in abundance/biomass. Empty cells indicate that the taxon was not present.*

| Taxon | Cat | Days after application | | | | | | | |
| --- | --- | --- | --- | --- | --- | --- | --- | --- | --- |
|  |  | -2 | 8 | 12 | 19 | 26 | 41 | 56 | 70 |
| a | 1 | ≥25 (67) | ≥25 (46) | ≥25 (66) | ≥25 (56) | ≥25 (52) | ≥25 (66) | ≥25 (53) | ≥25 (52) |
| b | 1 | ≥25 (61) | ≥25 (58) | ≥25 (65) | 7.5↓ (82) | ≥25 (90) | ≥25 (77) | ≥25 (67) | 15↑ (67) |
| c | 1 | ≥25 (44) | 5↓ (71) | 5↓ (58) | 7.5↓ (45) | 15↓ (71) | ≥25 (71) | 15↓ (70) | ≥25 (90) |
| d | 1 | - | ≥25 (69) | ≥25 (86) | ≥25 (82) | ≥25 (73) | ≥25 (57) | 15↓ (75) | - |
| e | 1 | ≥25 (83) | - | ≥25 (88) | ≥25 (55) | <1↑ (73) | ≥25 (80) | ≥25 (79) | - |
| f | 1 | 15↑ (339) | - | ≥25 (84) | ≥25 (73) | 5↓ (48) | ≥25 (51) | 5↓ (43) | 5↓ (51) |
| g | 1 | - | - | ≥25 (86) | ≥25 (89) | ≥25 (87) | ≥25 (70) | ≥25 (68) | ≥25 (72) |
| h | 1 | ≥25 (77) | ≥25 (69) | ≥25 (93) | ≥25 (99) | - | ≥25 (74) | ≥25 (77) | ≥25 (80) |
| i | 2 | - | - | - | 7.5↑ (284) | - | - | 15↑ (113) | - |
| j | 2 | - | 15↑ (132) | - | - | - | - | - |  |
| k | 2 | - | ≥25 (113) | 5↓ (62) | ≥25 (86) | - | - | - | 15↑ (n.c.) |
| l | 2 | - | 15↑ (n.c.) | - | - | - |  |  |  |
| m | 2 | - | - | - | - | ≥25 (81) | ≥25 (90) | ≥25 (96) | 7.5↑ (70) |
| n | 2 | - | 15↑ (n.c.) |  | - |  | - | - | - |
| o | 2 | - | - | 15↑ (n.c.) |  |  | - | - | - |
| p | 2 | ≥25 (66) | ≥25 (74) | 15↑ (61) | ≥25 (91) | ≥25 (91) | - | - | - |
| q | 2 | - | - | 15↑ (n.c.) | - | - | 15↑ (n.c.) | - | - |
| r | 3 | 5↑ (n.c.) | - | - | - | - | - | - | - |
| s | 3 | - | - | - | - | ≥25 (87) | - | - | - |
| t | 3 | - |  |  |  |  |  |  |  |
| u | 3 | - | - | - | - | - | - | - | - |
| v | 3 | - | - | - | - | - | - | - | - |
| w | 3 | - | ≥25 (99) | - | - | - | - | - | - |
| x | 3 | - |  | - |  |  |  |  |  |
| y | 3 | - | - | - | - | - | - | - | - |
| z | 3 | ≥25 (39) | ≥25 (83) | ≥25 (61) | ≥25 (98) | - | - | - | - |
